# Parameter Estimation Methods for Chaotic Intercellular Networks

**DOI:** 10.1371/journal.pone.0079892

**Published:** 2013-11-25

**Authors:** Inés P. Mariño, Ekkehard Ullner, Alexey Zaikin

**Affiliations:** 1 Departamento de Física, Universidad Rey Juan Carlos, Móstoles, Madrid, Spain; 2 Department of Physics, Institute for Complex Systems and Mathematical Biology and Institute of Medical Sciences, University of Aberdeen, Aberdeen, United Kingdom; 3 Institute for Women Health and Department of Mathematics, University College London, London, United Kingdom; 4 Department of Mathematics, King Abdulaziz University, Jeddah, Saudi Arabia; Humboldt University, Germany

## Abstract

We have investigated simulation-based techniques for parameter estimation in chaotic intercellular networks. The proposed methodology combines a synchronization–based framework for parameter estimation in coupled chaotic systems with some state–of–the–art computational inference methods borrowed from the field of computational statistics. The first method is a stochastic optimization algorithm, known as accelerated random search method, and the other two techniques are based on approximate Bayesian computation. The latter is a general methodology for non–parametric inference that can be applied to practically any system of interest. The first method based on approximate Bayesian computation is a Markov Chain Monte Carlo scheme that generates a series of random parameter realizations for which a low synchronization error is guaranteed. We show that accurate parameter estimates can be obtained by averaging over these realizations. The second ABC–based technique is a Sequential Monte Carlo scheme. The algorithm generates a sequence of “populations”, i.e., sets of randomly generated parameter values, where the members of a certain population attain a synchronization error that is lesser than the error attained by members of the previous population. Again, we show that accurate estimates can be obtained by averaging over the parameter values in the last population of the sequence. We have analysed how effective these methods are from a computational perspective. For the numerical simulations we have considered a network that consists of two modified repressilators with identical parameters, coupled by the fast diffusion of the autoinducer across the cell membranes.

## Introduction

Most dynamical systems studied in the physical, biological and social sciences that exhibit a rich dynamical behavior can be modeled by sets of nonlinear differential equations. These mathematical models are a useful tool to predict complex behaviors using numerical simulations. However, for the vast majority of systems, and particularly for biological systems, we lack a reliable description of the parameters of the model. In this paper we are interested in parameter estimation for coupled intercellular networks displaying chaotic behavior, since models of spontaneously active neural circuits typically exhibit chaotic dynamics (for example, spiking models of spontaneous activity in cortical circuits [1]–[3] and the analogous spontaneously active firing-rate model networks [4], [5]).

The problem of parameter estimation can be tackled in different ways, e.g., using multiple shooting methods [6]–[8] or some statistical procedures based on time discretizations and other approximations [9]–[13]. These methods involve the solution of high-dimensional minimization problems, since not only the unknown parameters but also the initial values of the trajectory segments between the sampling times need to be estimated [7], [14]. This is specially difficult when working with chaotic systems since very complicated error landscapes with many local minima can appear. In particular, notice that chaotic systems have an exponential sensitivity to initial conditions, that is, completely different trajectories can be obtained for identical parameter values and very similar initial conditions of the system variables.

On the other hand, several authors have suggested to take advantage of synchronization techniques for coupled chaotic systems and turn them into accurate parameter estimation methods [14]–[29]. There is a variety of techniques that rely on the synchronization properties of chaotic systems in order to tackle the parameter estimation problem. For example, some authors have proposed to handle the parameters as additional variables whose dynamics is described by tailored differential equations designed to have a fixed point at the true parameter values [14]–[20]. Adaptive estimation techniques relying on the time discretization of the state trajectories [22], [23], [30] and Monte Carlo methods [29], [31], including particle filters [32], [33], have also been investigated. All these techniques address the estimation of the system parameters independently of the initial conditions of the dynamic variables.

In this paper we investigate techniques that combine a synchronization–based framework for parameter estimation in coupled chaotic systems with some state–of–the–art computational inference methods borrowed from the recent literature in computational statistics. In particular, we describe estimation methods based on the accelerated random search (ARS) optimization algorithm [29], [31], [34] and two approximate Bayesian computation (ABC) [35]–[39] schemes. ABC is a general methodology for non–parametric inference that can be applied to practically any system of interest. We investigate two ABC–based methods. The first one is a Markov Chain Monte Carlo (MCMC) scheme [37] that generates a series of random parameter realizations for which a low synchronization error is guaranteed. We show that accurate parameter estimates can be obtained by averaging over these realizations. The second ABC scheme is termed Sequential Monte Carlo (SMC) ABC [38]. It generates a sequence of “populations”, i.e., sets of randomly generated parameter values, where the members of the 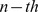 population attain a synchronization error that is lesser than the error attained by members of the 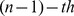 population. Again, we show how very accurate estimates can be obtained by averaging over the parameter values in the last population of the sequence. For the numerical simulations we consider a network of two coupled repressilators, since the repressilator is a paradigmatic gene regulatory system.

## Methods

### Structure of the Model

The repressilator is a prototype of a synthetic genetic clock built by three genes and the protein product of each gene represses the expression of another in a cyclic manner [40]. It can be constructed experimentally and produce near harmonic oscillations in protein levels. In the original repressilator design [40], the gene *lacI* expresses protein LacI, which inhibits transcription of the gene *tetR*. The product of the latter, TetR, inhibits transcription of the gene 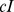. Finally, the protein product CI of the gene *cI* inhibits expression of *lacI* and completes the cycle. (See left-hand module in Fig. 1 of Ref. [41]).

**Figure 1:**
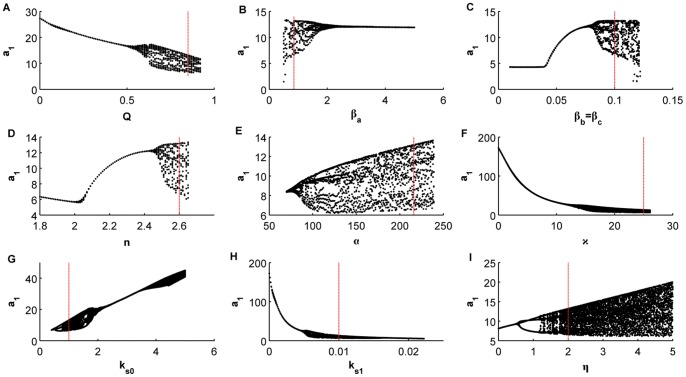
Bifurcation diagrams versus the different control parameters. (A) Q, (B) 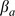, (C ) 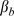, (D) 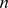, (E) 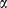, (F) 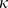, (G) 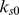, (H) 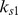, (I) *η*.

Cell-to-cell communication was introduced to the repressilator by García-Ojalvo and coworkers [42] by introducing an additional feedback loop to the network scheme that is based on the quorum sensing mechanism. The additional genetic module, which might be placed on a separate plasmid involves two other proteins [42]–[44]: LuxI, which produces a small autoinducer (AI) molecule that can diffuse through the cell membrane, and 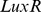, which responds to the autoinducer by activating transcription of a second copy of the repressilator gene *lacI*. The additional quorum sensing feedback loop can be connected to the basic repressilator in such a way that it reinforces the oscillations of the repressilator or competes with the overall negative feedback of the basic repressilator. The first one leads to phase attractive coupling for a robust synchronised oscillation [42] whereas the latter one evokes phase-repulsive influence [45]–[47], which is the key to multi-stability and a very rich dynamics including chaotic oscillations [41], [48], [49]. Placing the gene *luxI* under inhibitory control of the repressilator protein TetR (see Fig. 1 bottom in Ref. [41]) leads to the desired repressive and phase-repulsive coupling. We term this system modified repressilator. Phase repulsive coupling is common in several biological systems, e.g. in neural activity in the brain of songbirds [50], in the respiratory system [51], in the jamming avoidance response in electrical fish [52] and in the morphogenesis in Hydra regeneration and animal coat pattern formation [53].

**Figure 2:**
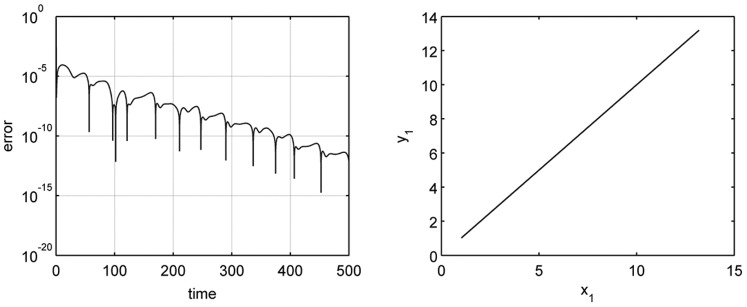
Synchronization between the primary and secondary systems. (left) Synchronization error, 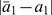, between both systems for 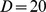. (right) The first variable of the secondary system (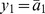) versus the first variable of the primary system (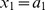).

In particular, in this work we are going to consider two modified repressilators with identical parameters, coupled by the fast diffusion of the autoinducer (AI) across the cell membranes. The mRNA dynamics is described by the following Hill-type kinetics with Hill coefficient *n*:
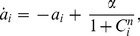
(1)

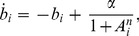
(2)

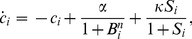
(3)where the subscript 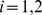 specifies the cell, and 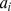, 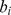, and 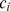 represent the concentrations of mRNA molecules transcribed from the genes of *tetR*, *cI*, and *lacI*, respectively. The parameter 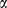 is the dimensionless transcription rate in the absence of a repressor. The parameter 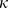 is the maximum transcription rate of the LuxR promoter.

The protein dynamics is given by
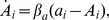
(4)

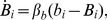
(5)

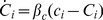
(6)where variables 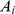, 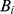, and 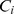 denote the concentration of the proteins TetR, CI, and LacI, respectively. The dynamics of the proteins is linked to the amount of the responsible mRNA, and the parameters 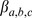 describe the ratio between mRNA and the protein lifetimes (inverse degradation rates). In what follows, we are going to assume 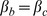. Thus, we can consider it as a single parameter. The model is made dimensionless by measuring time in units of the mRNA lifetime (assumed equal for all genes) and the mRNA and protein levels in units of their Michaelis constant. The mRNA concentrations are additionally rescaled by the ratio of their protein degradation and translation rates [42].

The third term on the right-hand side of Eq. (3) represents activated production of *lacI* by the AI molecule, whose concentration inside cell *i* is denoted by 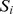. The dynamics of CI and LuxI can be considered identical, assuming equal lifetimes of the two proteins and given that their production is controlled by the same protein (TetR). Hence, the synthesis of the AI 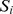 can be considered to be controlled by the concentration 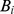 of the protein CI. Taking also into account the intracellular degradation of the AI and its diffusion toward or from the intercellular space, the dynamics of 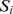 is given by
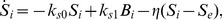
(7)where the diffusion coefficient *η* depends on the permeability of the membrane to the AI. Because of the fast diffusion of the extracellular AI (*S_e_*) compared to the repressilator period, we can apply the quasi-steady-state approximation to the dynamics of the external AI [42], which leads to



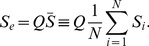
(8)The parameter *Q* is defined as
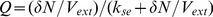
(9)where 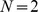 is the number of cells, 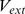 is the total extracellular volume, 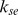 is the extracellular AI degradation rate, and 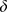 is the product of the membrane permeability and the surface area.

We achieve chaotic behavior for the following parameter values [41]: 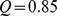, 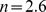, 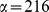, 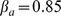, 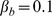, 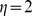, 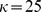, 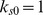, and 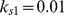. In particular, these are the values we are considering throughout this manuscript in order to assess the parameter estimation algorithms.

To see how the system behaves around these values we have plotted some bifurcation diagrams taking in each one a different parameter as a control parameter, whereas the rest of the parameters remain constant on the values mentioned above. Figure 1(A–I) represents the bifurcation diagrams versus the different control parameters. In particular, in each plot we are representing all maxima of the variable 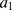 for some fixed initial condition as a function of the corresponding control parameter. We can observe how for some values of the control parameters the behavior changes from periodic to chaotic and vice versa. The vertical red dashed lines correspond to the values we are considering to assess the parameter estimation algorithms. Notice how for these values the system can display chaotic behavior.

### Problem Statement

We first introduce the notation to be used in the description of the parameter estimation methodologies. Let
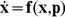
(10)represent the chaotic intercellular network (consisting of two coupled modified repressilators), with state variables
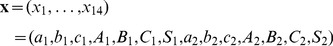
(11)and unknown parameters



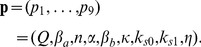
(12)The vector–valued function **f** can be explicitly written down by comparing Eq. (10) with Eqs. (1)-(7). In the sequel we refer to the system of Eq. (10) as *primary* system.

Since **f** in Eq. (10) is known, we can build a model with identical functional form but adjustable parameters and a coupling term, termed *secondary* system in the sequel, as
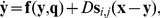
(13)where
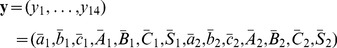
(14)is the time-varying vector that contains the model state variables,
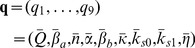
(15)is the adjustable parameter vector, D is a coupling coefficient and 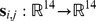 is a vector function that selects the i-th and the j-th element of its argument, i.e.,



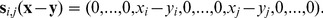
(16)The definition of the latter function implies that coupling is performed only through two scalar time series, 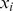 and 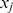, from the primary system. In particular, the coupling scheme we have chosen for the simulation setup in this paper is
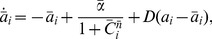
(17)where 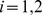 denotes the cell number (i.e., the repressilator index). Thus, the coupling only appears in two of the fourteen differential equations we have in the secondary system.

Since we assume identical functional form for the primary and secondary systems, when the secondary parameter vector, 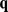, coincides with the primary parameter vector, 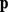, the state variables 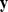 synchronize with 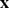 for 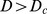, where 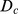 is a coupling threshold [29]. On the contrary, if 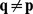 then identical synchronization cannot occur. However, the difference 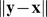 is expected to be small whenever the difference of the two parameter vectors is small and 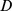 is sufficiently large. Therefore, the objective of a parameter estimation method based on synchronization is to compute a value 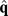 such that 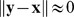 over time, since the latter implies 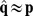.

Figure 2(left) shows how the synchronization error 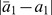 evolves in time when considering 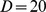 and identical parameters for the primary and the secondary systems. We can see how this error can be very small, less that 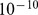. We are going to fix 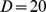 in all simulations throughout the paper. In Fig. 2(right) we are representing one variable of the secondary system (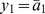) versus the same variable of the primary system (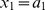) and observing the typical straight line, characteristic of the synchronization phenomenon.

It has been verified by means of numerical simulations that in order to obtain synchronization at least two coupling variables are needed (excluding 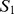 or 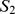), one from each repressilator in the primary system. Therefore, other combinations different from 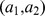 are also possible, as for example 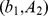. Coupling via a single variable is not sufficient to guarantee that the secondary system synchronizes with the primary one.

## Results

### Parameter Estimation Methods

Here we introduce three parameter estimation methods that combine the synchronization–based framework described above with state–of–the–art computational methods. In particular, we present results for the joint estimation of four parameters,
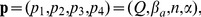
(18)in the coupled modified repressilator network. In all simulations shown in this manuscript we have assumed that just two scalar signals from the primary system are observed, namely the coupling variables 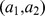, and we have numerically integrated the secondary system using a second order Runge-Kutta method with a time step 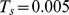 time units (t.u.).

### Accelerated Random Search

We first focus on a parameter estimation method for chaotic intercellular networks that takes advantage of chaos synchronization and is based on an efficient Monte Carlo optimization procedure, known as accelerated random search (ARS) method [29], [31]. In particular, the method we are going to describe consists in a variation of the technique proposed in [31]. Assuming that the variables 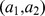 are observed during the time interval 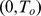, the value of the parameters in the secondary system can be calculated as the solution to the optimization problem
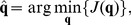
(19)where 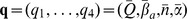 and the cost function
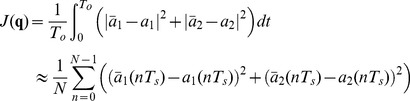
(20)is a quantitative representation of the synchronization error between the primary and the secondary systems. Notice that 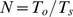, where 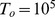 t.u.

The method can be outlined as follows:

1. Initialization. Choose:

(a) initial parameter values
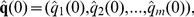
(21)where 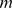 represents the number of parameters we want to estimate;

(b) maximum and minumum “search variances” for each parameter, 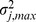 and 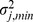, respectively, where 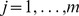;

(c) a “contraction factor” for each parameter, 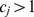, 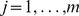.

Set 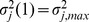 for 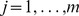 and, using the initial parameter vector, 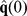, evaluate the associated cost 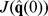.

2. *Iterative step.* Let 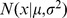 denote the Gaussian probability distribution of the variable 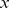 with mean 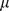 and variance 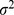. Assume we have computed a vector of parameter estimates, 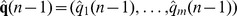, with cost 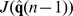.

Choose an index 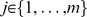, define
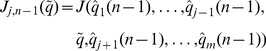
(22)and set 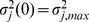 and 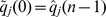. Choose an integer 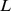 (the number of iterations to be performed over each parameter) and carry out the following computations:

For 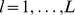:

The method can be outlined as follows:

1. Initialization. Choose:
  - initial parameter values
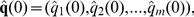
(21) where 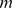 represents the number of parameters we want to estimate;
  - maximum and minumum “search variances” for each parameter, 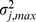 and 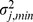, respectively, where 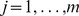;
  - a “contraction factor” for each parameter, 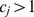, 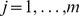.
  - Set 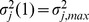 for 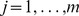 and, using the initial parameter vector, 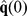, evaluate the associated cost 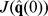.
2. *Iterative step.* Let 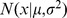 denote the Gaussian probability distribution of the variable 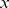 with mean 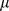 and variance 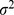. Assume we have computed a vector of parameter estimates, 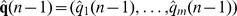, with cost 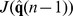.
  Choose an index 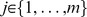, define
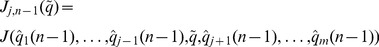
(22)

and set 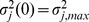 and 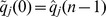. Choose an integer 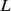 (the number of iterations to be performed over each parameter) and carry out the following computations:

For 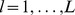:

- Draw 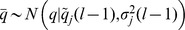.
- Compute 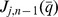.
- If 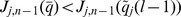 then
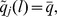


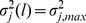
(23)else
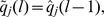


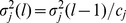
(24)
- If 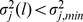, then
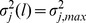
(25)

Once the loop is completed, set 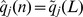 and 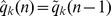 for every 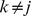.

Let 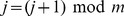, set 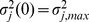 and perform the loop over (a)-(d) again.

The algorithm can be stopped when the synchronization error 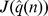 reaches a certain threshold or after a prescribed number of steps 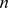 (e.g., when 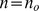 for some sufficiently large 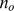). Notice that the total number of iterations is 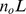 and the time evolution of the secondary system state, 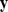, has to be integrated at each iteration, since a new candidate parameter vector is drawn each time.

We have carried out numerical simulations where this Monte Carlo optimization algorithm has been iterated a total of 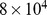 times, with the number of consecutive iterations for each parameter (iterations per loop) set to 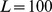, and the secondary system has been integrated for each iteration during 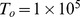 time units.

The initial values of the parameters are sampled from a uniform distribution, namely
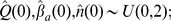


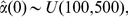
(26)the maximum search variances for each parameter are



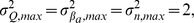


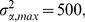
(27)the minimum search variances are



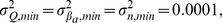


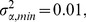
(28)and the contraction factors are



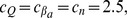


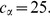
(29)


The normalized quadratic error for each parameter 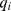, 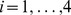, is defined as,
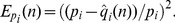
(30)


**Figure 3:**
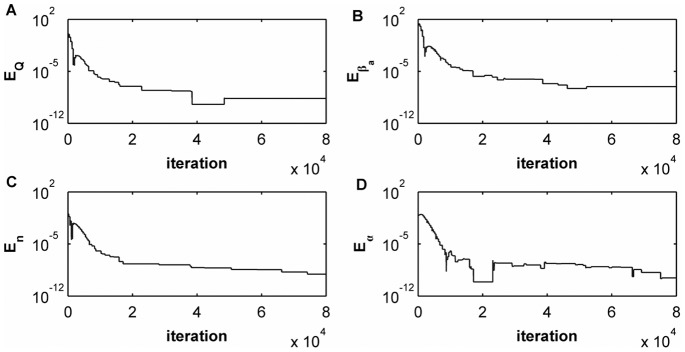
Normalized quadratic errors for each parameter as a function of the number of iterations for the ARS algorithm. (A) 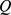, (B) 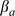, (C ) 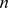 and (D) 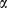.

Figure 3(A–D) shows the normalized quadratic errors for each parameter as a function of the number of iterations (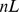). We can see how after 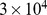 iterations all errors are very low for the estimated parameters. The values of the normalized quadratic errors in the 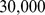-th iteration are:
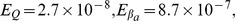


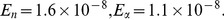
(31)


### Approximate Bayesian Computation

In Bayesian estimation theory the parameters are modeled as random variables, rather than unknown but deterministic numbers. Consequently, ABC-based methods aim at approximating the probability distribution of the parameters, vector 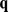, conditional on the observations from the primary system. The technique, to be described next, is nonparametric, i.e., the distribution of 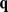 is approximated by a set of random samples.

ABC methods have been conceived with the aim of inferring posterior distributions without having to compute likelihood functions [35]–[39]. The calculation of the likelihood is replaced by a comparison between the observed and the simulated data. In the setup of this paper, the comparison is carried out between the data from the primary system (the observations) and the data generated by the secondary system (the model with adjustable parameters). The comparison between these data represents, in our case, a measure of the synchronization error between these two systems.

Let 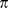 denote the a priori probability density function (pdf) of the random parameter vector 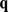, let 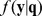 denote the probability distribution of the data 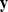 generated by the secondary system conditional on the realization of 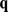 and let 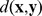 be a distance function that measures the synchronization error by comparing the observed time series 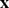 from the primary system and the generated time series 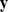 from the secondary one. Since the system of interest is deterministic, 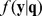 collapses into a Dirac delta measure when 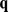 is given. In the ABC framework, though, we are not interested in evaluating 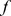 but rather in generating 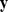 using the model conditional on 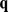. This amounts to integrating the secondary system with a given realization of the adjustable parameters 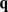.

The simplest ABC algorithm is the *ABC rejection sampler* [35]. For a given synchronization error threshold (often termed tolerance) 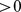, the algorithm can be described as follows.

1. Sample 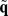 from 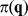.
2. Simulate a dataset 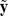 from the conditional probability distribution 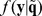.
3. If the distance function (synchronization error) is 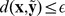, then accept 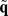, otherwise reject it.
4. Return to the first step.

The output of an ABC algorithm is a population of parameter values randomly drawn from the distribution 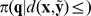, i.e., a distribution with density proportional to the prior but restricted to the set of values of 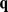 for which the synchronization error is at most .

The disadvantage of the ABC rejection sampler is that the acceptance rate is very low when the set of values of 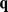 for which the synchronization error is less than  turns out to be relatively small. Thus, instead of implementing this method we have decided to implement two more sophisticated ABC algorithms. The first one is a Markov chain Monte Carlo (ABC MCMC) technique and the second one is a sequential Monte Carlo (ABC SMC) method.

### Approximate Bayesian Computation Markov Chain Monte Carlo

The Approximate Bayesian Computation Markov Chain Monte Carlo (ABC MCMC) algorithm is a Metropolis-Hastings [37] MCMC method that incorporates one additional test to ensure that all parameters in the chain yield a synchronization error below the threshold . It can be outlined as follows.

1. Initialize 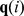, 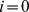.
2. Generate 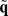 according to a proposal distribution 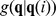

3. Simulate a dataset 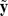 from the conditional probability distribution 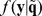.
4. If the distance function (synchronization error) is 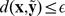, go to the following step, otherwise set 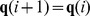 and go to step 6.
5. Set 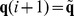 with probability

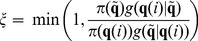
(32)and 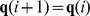 with probability 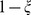.

1. Set 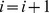, go to step 2.

The outcome of this algorithm is a Markov chain with the stationary distribution 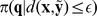 [37]. The parameters are assumed independent a priori, hence
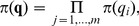
(33)and we also choose independent proposals for simplicity. In particular, the prior distributions for each parameter have been chosen as



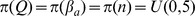


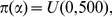
(34)and the proposal distribution for each parameter is a Gaussian distribution centered at the previous value of the corresponding parameter and with a fixed standard deviation, different for each parameter. In particular,



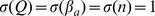


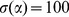
(35)and



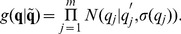
(36)Since the proposal distribution is symmetric, 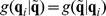, and the prior is uniform, the acceptance probability in Eq.(32) is 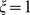.

The distance function (synchronization error) has been chosen as
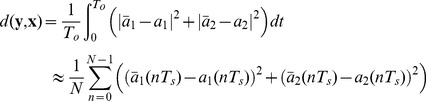
(37)where 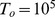 t.u. and 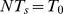. This expression is equivalent to the cost function in the ARS method. The tolerance (threshold for the synchronization error) has been chosen as 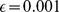.

**Figure 4:**
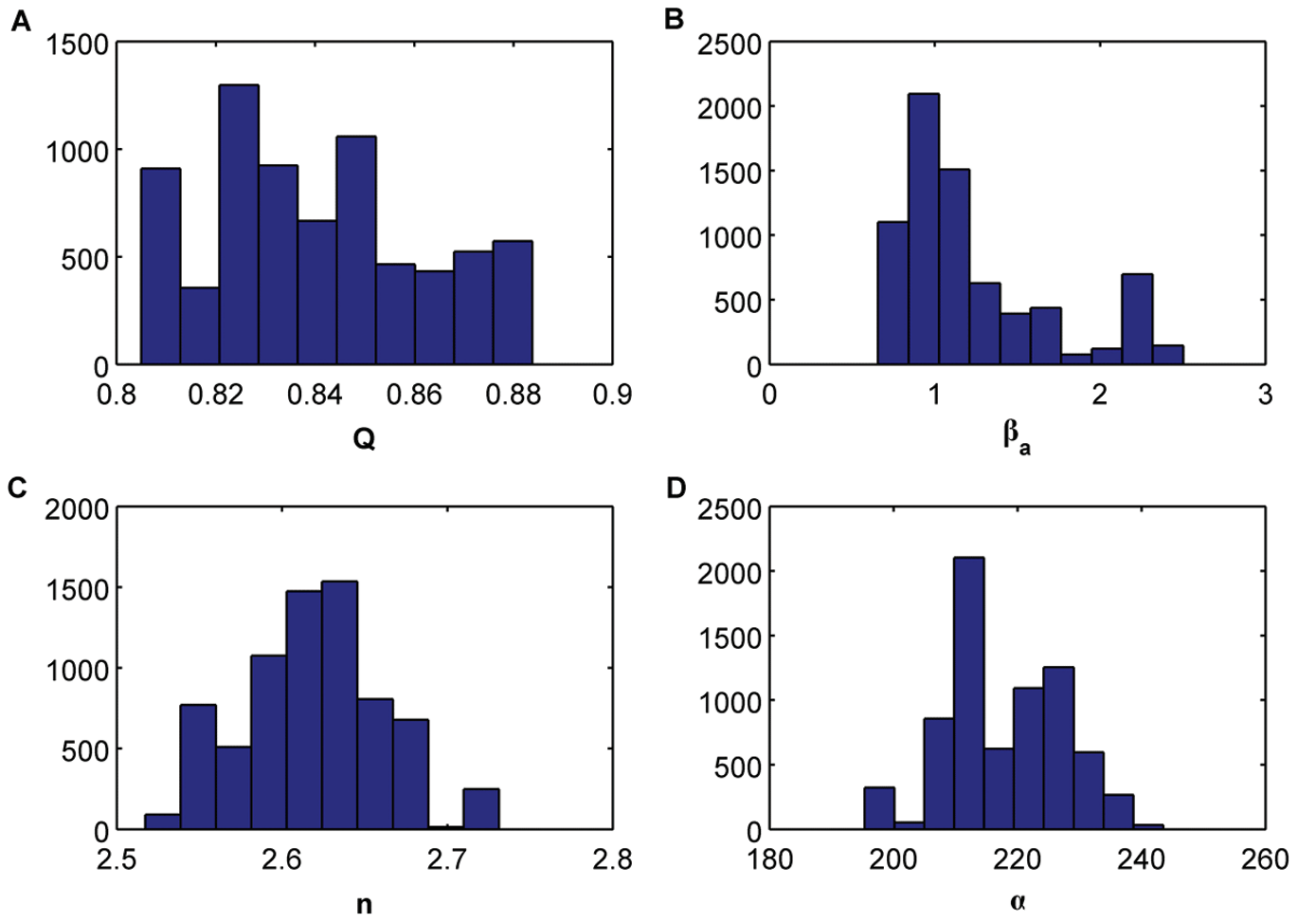
Histograms of the approximate marginal posterior distributions for each parameter for the ABC MCMC algorithm when considering 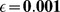. (A) 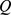, (B) 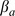, (C ) 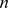 and (D) 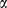.



A chain of 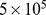 samples has been generated, what implies that the secondary system has been integrated 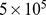 times. The initial point of the chain is selected to ensure that the associated distance is less than 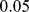. Figure 4(A–D) shows the histograms of the approximate marginal posterior distributions for each parameter. In order to reduce the strong correlation between consecutive samples in the Markov chain we have subsampled by a factor of 50. We have calculated the mean values of each histogram as well as the normalized quadratic errors according to the following expression
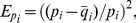
(38)where 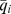, for 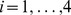, represents the mean value of the histogram of the corresponding parameter. The values of the normalized quadratic errors for the estimated parameters are

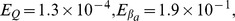


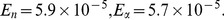
(39)


We can see how three parameters are accurately estimated whereas for one of them, 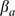 the error is significantly higher compared to (31).

### Approximate Bayesian Computation Sequential Monte Carlo

A more sophisticated application of the ABC methodology is the Approximate Bayesian Computation Sequential Monte Carlo algorithm [39], [54], [55]. In ABC SMC, a number of sampled parameter values (often termed particles), 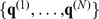, drawn from the prior distribution 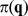, are propagated through a sequence of intermediate distributions 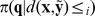, 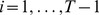, until they are converted into samples from the *target distribution* 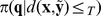. The tolerances are chosen such that 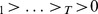, thus the empirical distributions gradually evolve towards the target posterior. The ABC SMC algorithm proceeds as follows [39].

1. Initialize 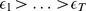. Set the population indicator 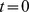.
2. Set the particle indicator 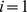.
3. If 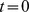, draw 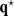 from the prior 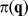.

Else, draw 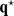 from
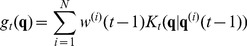
(40)where 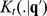 is a symmetric kernel centred around 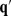 and 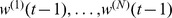 are importance weights such that 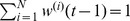.

1. If 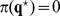, return to step 3.
2. Simulate a candidate dataset 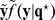.
3. If 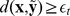 return to step 3.
4. Set 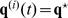 and calculate the weight,
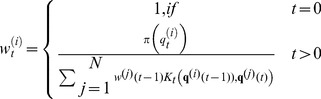
(41)
5. If 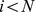, set 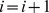 and go to step 3.
6. Normalize the weights.
7. If 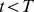, set 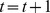 and go to step 2. Otherwise stop.

The prior distributions we have considered for each parameter are 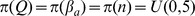 and 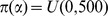, the same as for the ABC MCMC algorithm. The perturbation kernel 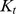 is Gaussian, namely



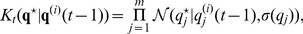
(42)with standard deviations 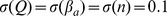 and 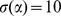. The distance function (synchronization error) is the same as for the ABC MCMC and ARS algorithms with the same 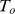 value. To ensure the gradual transition between populations, the ABC SMC algorithm is run for 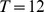 populations with 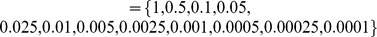 and we have considered 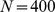 particles per population.

**Figure 5:**
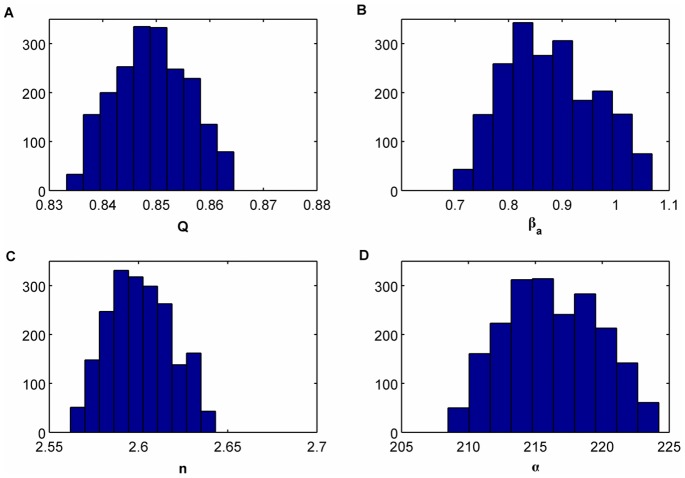
Histograms of the approximate marginal posterior distributions for each parameter for the ABC SMC algorithm when considering 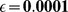. (A) 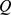, (B) 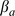, (C ) 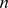 and (D) 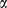.



Figure 5(A–D) shows the histograms of the approximate marginal posterior distributions for each parameter where the different weights of the different particles have been taken into account for the representation. We have calculated the mean values of each histogram (that match the actual values) as well as the normalized quadratic errors using Eq. (38), that is, the same expression as for the ABC MCMC algorithm. The values of the normalized quadratic errors for the estimated parameters with the ABC SMC method are

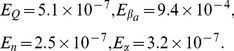
(43)


**Figure 6:**
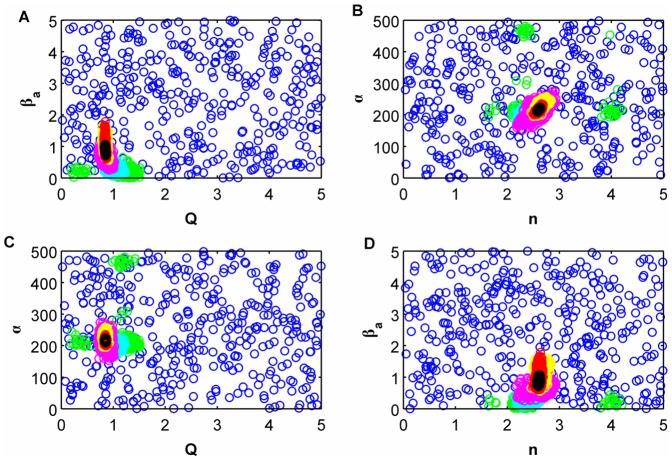
Two-dimensional scatterplots for the accepted particles of the ABC SMC of each population. The particles from the prior are represented in blue, particles from population 2 in green, particles from population 4 in cyan, population 6 in pink, 8 in yellow, 10 in red and particles from the posterior (population 12) in black color.

Figure 6(A–D) shows the output (i.e. the accepted particles) of the ABC SMC algorithm as scatterplots of some of the two-dimensional parameter combinations, where we have information of different populations in the same plot. As we iterate the algorithm we obtain populations that are more dense around the desired values, as shown in these plots, where the particles from the prior are represented in blue, particles from population 2 in green, particles from population 4 in light blue, population 6 in pink, 8 in yellow, 10 in red and particles from the posterior (population 12) in black color. We can see how for the last population the particles are tightly clustered around the desired value.

**Figure 7:**
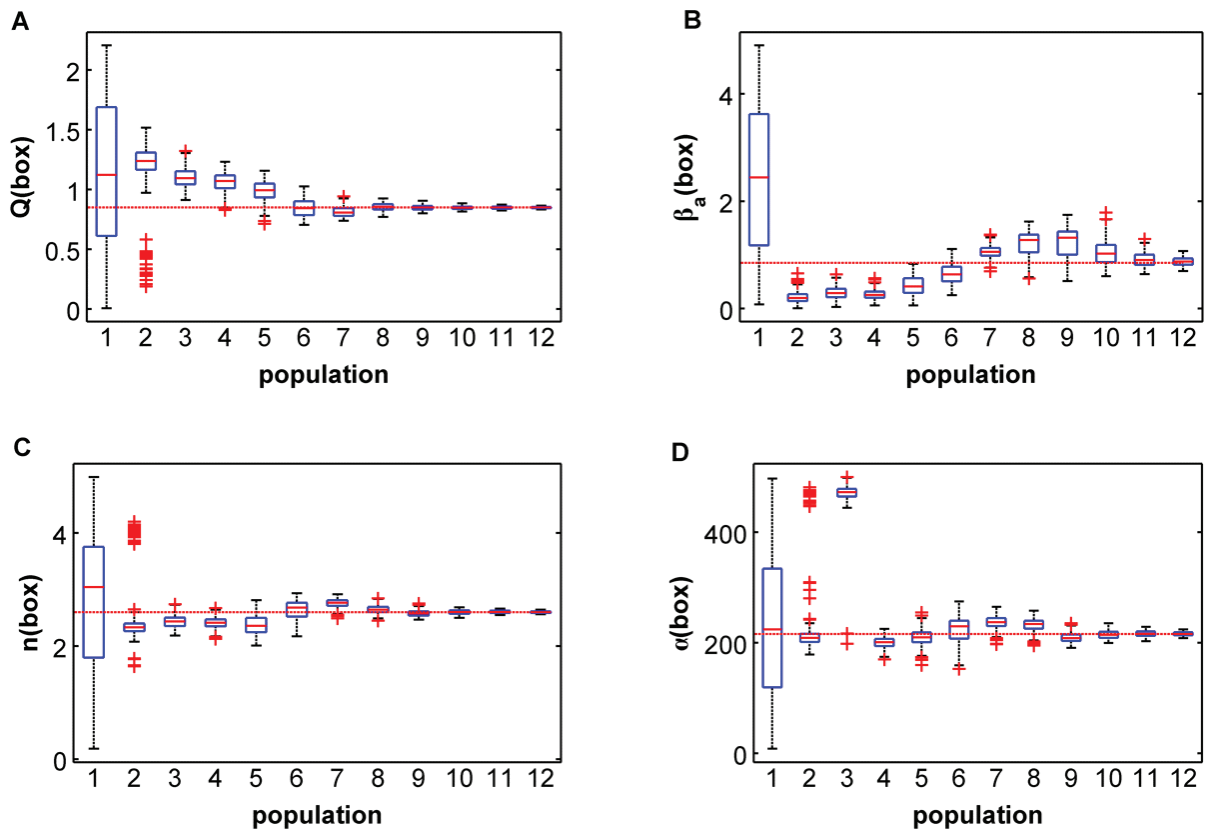
Box plots diagrams for the different populations for each parameter. (A) Q, (B) 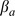, (C ) n and (D) 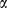.

In order to gain insight of how the parameters are estimated during the evolution of the algorithm, we have represented some box–plot diagrams, one for each parameter to be estimated, as seen in fig. 7(A–D). In each diagram, we have information about the corresponding parameter as a function of the population index. In particular, the central mark of each box is the median of the population, the edges of the box are the 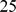-th and 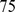-th percentiles, the whiskers extend to the most extreme data points not considered as outliers, and the outliers are plotted individually using the plus symbols in red. The horizontal lines in red represent the actual values of the parameters we have used in our simulation. We can see from these plots how for a high enough population index the median values of the four parameters perfectly match the actual values.

**Figure 8:**
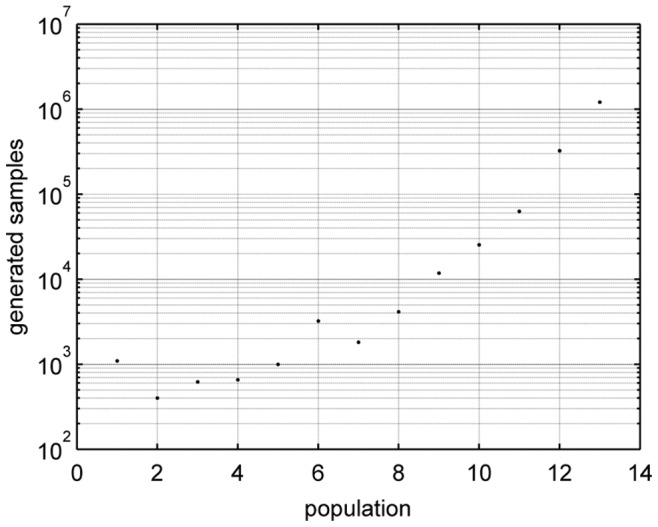
Computational cost for each population using the ABC SMC algorithm.

We have also studied the computational cost of the algorithm. In fig. 8 we can see the number of samples or particles we have generated in order to have 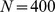 accepted particles for each population. We can see how the number of particles increases with the population index, being significantly high for the last population index, since it corresponds to a very small value of the synchronization error. Notice that the vertical axis of this figure is in a logarithmic scale.

### Comparison of the Methods

Here we compare not only the accuracy but also the computational complexity of all three Monte Carlo methods for the joint estimation of the four parameters, 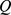, 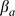, n and 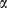, of the chaotic intercellular network. To do that, we have calculated for the ABC SMC algorithm the computational load up to each population. Specifically, the computational complexity of generating a sequence of 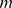 populations is given by the number of samples or particles that have to be generated before completing the 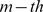 population. Note that the computational load for the 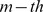 population also includes all samples needed to generate the 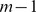 previous populations.

**Figure 9:**
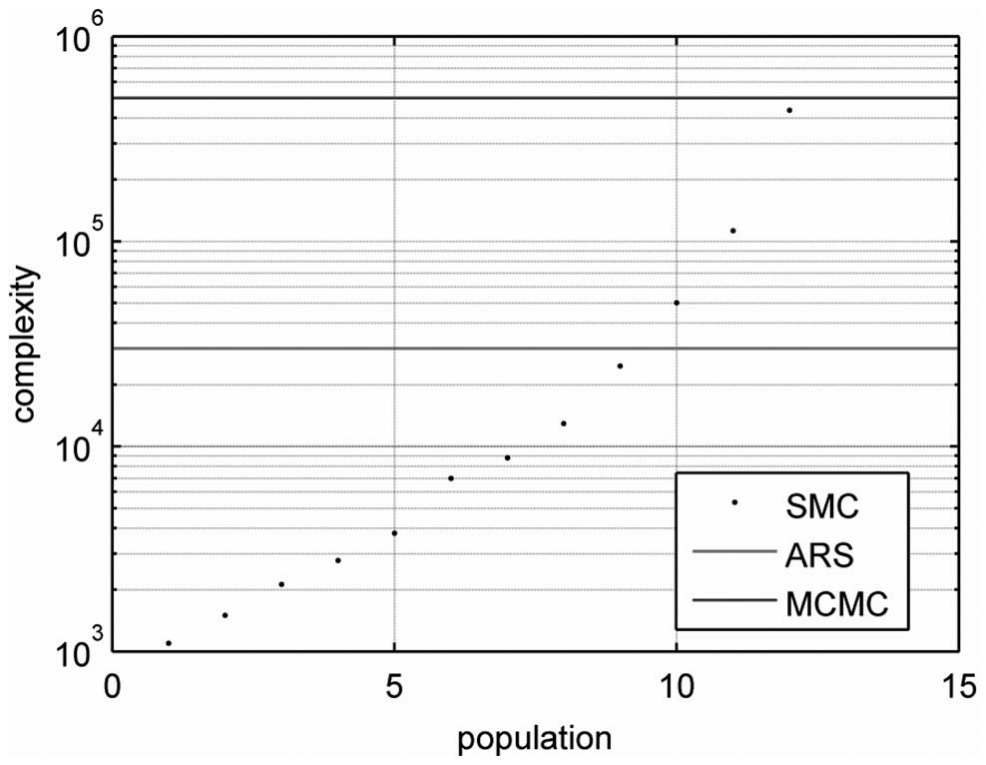
Computational complexity measured by the number of random samples generated by the algorithms. The solid blue line is the complexity of the ABC MCMC algorithm (with 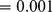). The solid red line is the complexity of the ARS method. The black dots indicate the complexity of the ABC SMC algorithm, for each population up to the 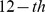 one.

Fig. 9 provides a graphical depiction of the complexity of the three methods, that we have investigated (ARS, ABC MCMC and ABC SMC algorithms). The line in red represents the complexity for the ARS method and the line in blue indicates the complexity for the ABC MCMC technique.

The parameter estimation errors attained with the ARS method are of the same order as the errors of the parameter estimates computed from the 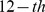 population of the ABC SMC algorithm. However, the number of samples generated to run the ARS procedure is 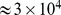 while the ABC SMC technique demands the generation of 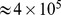 random samples up to the 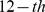 population. The ABC MCMC method achieves the poorest performance, as it requires the generation of the highest number of samples (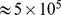) and produces the largest errors (up to three orders of magnitude worse than the ARS or ABC SMC estimates).

## Discussion

We have investigated three computational inference techniques for parameter estimation in a chaotic intercellular network that consists of two coupled modified repressilators. The proposed methodology combines a synchronization–based framework for parameter estimation in coupled chaotic systems with some state–of–the–art computational inference methods borrowed from computational statistics. In particular, we have focussed on an accelerated random search algorithm and two approximate Bayesian computation schemes (ABC MCMC and ABC SMC). The three methods exploit the synchronization property of chaotic systems. Therefore, it is not necessary to estimate the initial conditions of the variables, which is an important advantage from a computational point of view.

We have carried out the numerical study in this paper assuming that only two variables from the primary system can be observed. This is the minimum number of observed variables in order to guarantee the synchronization of the secondary system. If additional variables can be observed it is possible to easily incorporate them into the proposal methodology. For example, if the variables 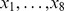 are observed (this is the full state of the first repressilator and the first variable, 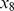, of the second repressilator) we can redefine the distance function of Eq. (38) as



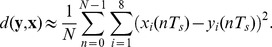
(44)


It can be verified (numerically) that using the distance in (44) (which intuitively provides “more information” about the primary system) leads to more accurate parameter estimates (or, alternatively, a greater number of parameters can be estimated if necessary). Note, however, that this comes at the expense of an additional computational effort and, moreover, it is unclear that all these variables can be accurately measured in practice.

The proposed methods can be applied when the observed time series are contaminated with additive noise of moderate variance. For example, if the observations have the form


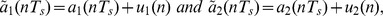
 where 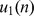 and 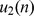 are sequences of independent and identically distributed Gaussian noise variables with zero mean and variance 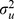, then the distance function of Eq. (30) is lower-bounded by the noise variance. Specifically, if 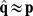 and, hence, we assume that 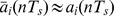, it turns out that the distance 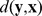 is an estimator of (twice) the noise variance



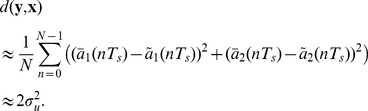
(45)


This indicates that the synchronization error cannot go below the (approximate) bound of 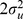 and, therefore, the ABC-based methods can work as long as the tolerances (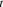, 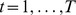, in the ABC SMC method, or 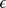 in the ABC MCMC technique) are chosen to be greater than 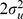. This means that the ABC SMC algorithm with 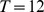 populations and 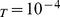 can still provide accurate parameter estimates when the observation noise variance is 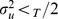. In order to handle larger noise variances, one needs to relax the coupling (i.e., choose a smaller coupling factor 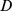) and increase the observation period 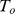. This makes the distance function more sensitive to the discrepancy between 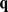 and 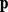, which in practice means that we can choose a larger tolerance (e.g, 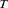 in the ABC SMC algorithm) and preserve the accuracy of the resulting estimate 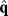.

## References

[1] van Vreeswijk C, Sompolinsky H (1996) Chaos in Neuronal Networks with Balanced Excitatory and Inhibitory Activity. Science 274: 1724–1726. 8939866 10.1126/science.274.5293.1724

[2] Amit DJ, Brunel N (1997) Model of global spontaneous activity and local structured activity during delay periods in the cerebral cortex. Cereb. Cortex 7: 237–252. 10.1093/cercor/7.3.237 9143444

[3] Brunel N (2000) Dynamics of networks of randomly connected excitatory and inhibitory spiking neurons. J. Physiol Paris 94: 445–463. 11165912 10.1016/s0928-4257(00)01084-6

[4] Sompolinsky H, Crisanti A, Sommers HJ (1988) Chaos in random neural networks. Phys. Rev. Lett. 61: 259–262. 10.1103/PhysRevLett.61.259 10039285

[5] Sussillo D, Abbott LF (2009) Generating Coherent Patterns of Activity from Chaotic Neural Networks. Neuron 63: 544–557. 19709635 10.1016/j.neuron.2009.07.018 PMC2756108

[6] Ghosh A, Kumar VR, Kulkarni BD (2001) Parameter estimation in spatially extended systems: The Karhunen-Leve and Galerkin multiple shooting approach. Phys. Rev. E 64: 056222. 10.1103/PhysRevE.64.056222 11736069

[7] Baake E, Baake M, Bock HG, Briggs KM (1992) Fitting ordinary differential equations to chaotic data. Phys. Rev. A 45: 5524–5529. 10.1103/physreva.45.5524 9907650

[8] Hatz K, Schloder JP, Bock HG (2012) Estimating Parameters in Optimal Control Problems. SIAM J. Sci. Comput. 34: A1707–A1728.

[9] Petridis V, Paterakis E, Kehagias A (1998) A hybrid neural-genetic multimodel parameter estimation algorithm. IEEE Trans. Neural Networks 9: 862–876. 18255772 10.1109/72.712158

[10] Timmer J (2000) Parameter estimation in nonlinear stochastic differential equations. Chaos Solitons and Fractals 11: 2571–2578.

[11] Singer H (2002) Parameter Estimation of Nonlinear Stochastic Differential Equations: Simulated Maximum Likelihood versus Extended Kalman Filter and It-Taylor Expansion. Journal of Computational and Graphical Statistics 11: 972–995.

[12] Sitz A, Schwarz U, Kurths J, Voss HU (2002) Estimation of parameters and unobserved components for nonlinear systems from noisy time series. Phys Rev. E 66: 016210. 10.1103/PhysRevE.66.016210 12241464

[13] Pisarenko VF (2004) SornetteD (2004) Statistical methods of parameter estimation for deterministically chaotic time series. Phys. Rev. E 69: 036122. 10.1103/PhysRevE.69.036122 15089376

[14] Parlitz U, Junge L, Kocarev L (1996) Synchronization-based parameter estimation from time series. Phys. Rev. E 54: 6253–6259. 10.1103/physreve.54.6253 9965845

[15] Parlitz U (1996) Estimating Model Parameters from Time Series by Autosynchronization. Phys. Rev. Lett. 76: 1232–1235. 10.1103/PhysRevLett.76.1232 10061669

[16] Zhou C, Lai C-H (1999) Decoding information by following parameter modulation with parameter adaptive control. Phys. Rev. E 59: 6629–6636. 10.1103/physreve.59.6629 11969650

[17] Maybhate A, Amritkar RE (1999) Use of synchronization and adaptive control in parameter estimation from a time series. Phys. Rev. E 59: 284–293.

[18] d’Anjou A, Sarasola C, Torrealdea FJ, Orduna R, Grana M (2001) Parameter-adaptive identical synchronization disclosing Lorenz chaotic masking. Phys. Rev. E 63: 046213. 10.1103/PhysRevE.63.046213 11308936

[19] Konnur R (2003) Synchronization-based approach for estimating all model parameters of chaotic systems. Phys. Rev. E 67: 027204. 10.1103/PhysRevE.67.027204 12636863

[20] Huang D (2004) Synchronization-based estimation of all parameters of chaotic systems from time series. Phys. Rev. E 69: 067201. 10.1103/PhysRevE.69.067201 15244789

[21] Freitas US, Macau EEN, Grebogi C (2005) Using geometric control and chaotic synchronization to estimate an unknown model parameter. Phys. Rev. E 71: 047203. 10.1103/PhysRevE.71.047203 15903824

[22] Mariño IP, Míguez J (2005) Adaptive approximation method for joint parameter estimation and identical synchronization of chaotic systems. Phys. Rev. E 72: 057202. 10.1103/PhysRevE.72.057202 16383795

[23] Mariño IP, Míguez J (2006) An approximate gradient-descent method for joint parameter estimation and synchronization of coupled chaotic systems. Phys. Lett. A 351: 262–267.

[24] Tao C, Zhang Y, Jiang JJ (2007) Estimating system parameters from chaotic time series with synchronization optimized by a genetic algorithm. Phys. Rev. E 76: 016209. 10.1103/PhysRevE.76.016209 17677545

[25] Yang X, Xu W, Sun Z (2007) Estimating model parameters in nonautonomous chaotic systems using synchronization. Phys. Lett. A 364: 378–388.

[26] Yu D, Parlitz U (2008) Estimating parameters by autosynchronization with dynamics restrictions. Phys. Rev. E 77: 066221. 10.1103/PhysRevE.77.066221 18643364

[27] Ghosh D, Banerjee S (2008) Adaptive scheme for synchronization-based multiparameter estimation from a single chaotic time series and its applications. Phys. Rev. E 78: 056211. 10.1103/PhysRevE.78.056211 19113204

[28] Abarbanel HDI, Creveling DR, Farsian R, Kostuk M (2009) Dynamical State and Parameter Estimation. SIAM J. Appl. Dyn. Syst. 8: 1341–1381.

[29] Sakaguchi H (2002) Parameter evaluation from time sequences using chaos synchronization. Phys. Rev. E 65: 027201. 10.1103/PhysRevE.65.027201 11863696

[30] Schumann-Bischoff J, Luther S, Parlitz U (2013) Nonlinear system identification employing automatic differentiation. Commun. Nonlinear Sci. Numer. Simulat. 18: 2733–2742.

[31] Mariño IP, Míguez J (2007) Monte Carlo method for multiparameter estimation in coupled chaotic systems. Phys. Rev. E 76: 057203. 10.1103/PhysRevE.76.057203 18233798

[32] van Leeuwen PJ (2010) Nonlinear data assimilation in geosciences: an extremely efficient particle filter. Q.J.R. Meteorol. Soc 136: 1991–1999.

[33] Mariño IP, Míguez J, Meucci R (2009) Monte Carlo method for adaptively estimating the unknown parameters and the dynamic state of chaotic systems. Phys. Rev. E 79: 056218. 10.1103/PhysRevE.79.056218 19518547

[34] Appel MJ, Labarre R, Radulovic D (2003) On Accelerated Random Search. SIAM J. Optim. 14: 708–731.

[35] Pritchard J, Seielstad MT, Perez-Lezaun A, Feldman MW (1999) Population growth of human Y chromosomes: a study of Y chromosome microsatellites. Mol. Biol. Evol. 16: 1791–1798. 10.1093/oxfordjournals.molbev.a026091 10605120

[36] Beaumont MA, Zhang W, Balding DJ (2002) Approximate Bayesian Computation in Population Genetics. Genetics 162: 2025–2035. 12524368 10.1093/genetics/162.4.2025 PMC1462356

[37] Marjoram P, Molitor J, Plagnol V, Tavare S (2003) Markov chain Monte Carlo without likelihood. Proc. Natl. Acad. Sci. U.S.A. 100: 15324–15328. 10.1073/pnas.0306899100 PMC307566 14663152

[38] Sisson SA, Fan Y, Tanaka MM (2007) Sequential Monte Carlo without likelihoods. Proc. Natl. Acad. Sci U.S.A. 104: 1760–1765. 10.1073/pnas.0607208104 PMC1794282 17264216

[39] Toni T, Welch D, Strelkowa N, Ipsen A, Stumpf MPH (2009) Approximate Bayesian computation scheme for parameter inference and model selection in dynamical systems. J. R. Soc. Interface 6: 187–202. 19205079 10.1098/rsif.2008.0172 PMC2658655

[40] Elowitz MB, Leibler S (2000) A synthetic oscillatory network of transcriptional regulators. Nature 403: 335–338. 10659856 10.1038/35002125

[41] Ullner E, Koseka A, Kurths J, Volkov E, Kantz H, et al (2008) Multistability of synthetic genetic networks with repressive cell-to-cell communication. Phys. Rev. E 78: 031904. 10.1103/PhysRevE.78.031904 18851062

[42] García-Ojalvo J, Elowitz B, Strogatz SH (2004) Modeling a synthetic multicellular clock: Repressilators coupled by quorum sensing. Proc. Natl. Acad. Sci. U.S.A. 101: 10955–10960. 10.1073/pnas.0307095101 PMC503725 15256602

[43] McMillen D, Kopell N, Hasty J, Collins JJ (2002) Synchronizing genetic relaxation oscillators by intercell signaling. Proc. Natl. Acad. Sci. U.S.A. 99: 679–684. 10.1073/pnas.022642299 PMC117365 11805323

[44] You L, Cox III RS, Weiss R, Arnold FH (2004) Programmed population control by cellcell communication and regulated killing. Nature 428: 868–871. 15064770 10.1038/nature02491

[45] Volkov EI, Stolyarov MN (1991) Birhythmicity in a system of two coupled identical oscillators. Phys. Lett. A 159, 61–66.

[46] Han SK, Kurrer C, Kuramoto Y (1995) Dephasing and Bursting in Coupled Neural Oscillators. Phys. Rev. Lett. 75: 3190–3193. 10.1103/PhysRevLett.75.3190 10059517

[47] Balázsi G, Cornell-Bell A, Neiman AB, Moss F (2001) Synchronization of hyperexcitable systems with phase-repulsive coupling. Phys. Rev. E 64: 041912. 10.1103/PhysRevE.64.041912 11690057

[48] Ullner E, Zaikin A, Volkov EI, García-Ojalvo J (2007) Multistability and Clustering in a Population of Synthetic Genetic Oscillators via Phase-Repulsive Cell-to-Cell Communication. Phys. Rev. Lett. 99: 148103. 10.1103/PhysRevLett.99.148103 17930726

[49] Koseka A, Ullner E, Volkov E, Kurths J, García-Ojalvo J (2010), J. Theor. Biol. 263, 189. 10.1016/j.jtbi.2009.11.007 19932703

[50] Laje R, Mindlin GB (2002)., Phys. Rev. Lett. 89, 288102. 10.1103/PhysRevLett.89.288102 12513182

[51] Koseska A, Volkov E, Zaikin A, Kurths J (2007) Inherent multistability in arrays of autoinducer coupled genetic oscillators. Phys. Rev. E 75: 031916. 10.1103/PhysRevE.75.031916 17500735

[52] Glass L, Mackey MC (1988) From Clocks to Chaos: The Rhythms of Life: Princeton University Press, Princeton, NJ. 248p.

[53] Meinhardt H (1982) Models of Biological Pattern Formation: Academic Press, New York.

[54] Del Moral P, Doucet A, Jasra A (2006) Sequential Monte Carlo samplers. J. R. Stat. Soc B 68: 411–436.

[55] Del Moral P, Doucet A, Jasra A (2012) An adaptive sequential Monte Carlo method for approximate Bayesian computation. Stat Comput 22: 1009–1020.

